# Rate‐responsive pacing and atrial high rate episodes in cardiac resynchronization therapy patients: Is low heart rate the key?

**DOI:** 10.1002/clc.23227

**Published:** 2019-07-07

**Authors:** Mauro Biffi, Antonio D'Onofrio, Carlo Pignalberi, Ennio C. Pisanò, Saverio Iacopino, Antonio Curnis, Gaetano Senatore, Alessandro Capucci, Paolo Della Bella, Valeria Calvi, Gabriele Zanotto, Fabrizio Caravati, Giampiero Maglia, Michele Manzo, Matteo Santamaria, Matteo Ziacchi, Fabio Lissoni, Daniele Giacopelli, Alessio Gargaro, Francesco Solimene

**Affiliations:** ^1^ Policlinico Sant'Orsola‐Malpighi Bologna Italy; ^2^ Ospedale Monaldi Naples Italy; ^3^ Ospedale San Filippo Neri Rome Italy; ^4^ Ospedale Vito Fazzi Lecce Italy; ^5^ Villa Maria Care and Research Cotignola Italy; ^6^ Spedali Civili Brescia Italy; ^7^ Ospedale di Ciriè Ciriè (TO); ^8^ Ospedali Riuniti Ancona Italy; ^9^ Ospedale San Raffaele Milan Italy; ^10^ Policlinico Vittorio Emanuele PO Ferrarotto Catania Italy; ^11^ Ospedale Mater Salutis Legnago Italy; ^12^ ASST Settelaghi‐Ospedale di Circolo‐ Varese Italy; ^13^ Azienda Ospedaliera Pugliese Ciaccio Catanzaro; ^14^ Azienda Ospedaliera Universitaria S.Giovanni di Dio e Ruggi D'Aragona Salerno Italy; ^15^ Fondazione di Ricerca e Cura Giovanni Paolo II Campobasso Italy; ^16^ Ospedale Maggiore di Lodi Lodi Italy; ^17^ BIOTRONIK Italia Vimodrone (MI) Italy; ^18^ Clinica Montevergine Mercogliano (AV) Italy

**Keywords:** atrial arrhythmias, atrial pacing, cardiac resynchronization therapy, heart failure, rate‐responsiveness

## Abstract

**Background:**

The role of atrial rate‐responsive (RR) pacing in cardiac resynchronization therapy (CRT) is unclear due to the favorable effect of rate lowering in systolic heart failure. Atrial high rate episodes (AHREs) in CRT recipients are particularly worrisome since they cause loss of CRT, beyond representing a stroke risk factor.

**Hypothesis:**

The presence of an association between RR and the incidence of AHREs.

**Methods:**

Daily remote transmissions from 836 CRT recipients were analyzed. AHREs were classified by duration: ≥15 minutes, ≥5 hours, and ≥ 24 hours. Variables possibly associated to AHREs were included in time‐dependent proportional‐hazard models, averaging over 30‐day periods and adjusting for main baseline variables.

**Results:**

After a median follow‐up of 23.9 (12.2‐36.0) months, 507 (60.6%) patients experienced at least one 15‐minute AHRE. RR function was programmed in 166 (19.8%) patients and was associated with an increased AHRE occurrence rate with hazard ratio (HR) ranging from 1.45 to 1.78 for the 3 cutoffs of episode duration. The negative effect of RR function was not observed in the subset of patients with low mean heart rate (<68 bpm). Higher mean heart rates increased AHRE risk (HR:1.02, *P* = .01), while CRT amount decreased it (HR:0.98, *P* < .01). The extent of atrial pacing did not predict AHRE occurrence.

**Conclusions:**

RR pacing in CRT recipients is associated with increased AHRE occurrence, especially when an average heart rate > 68 bpm is attained.

## 1 INTRODUCTION

Cardiac resynchronization (CRT) is a well‐established therapy for symptomatic heart failure (HF) patients with systolic dysfunction and prolonged QRS.^1^, ^2^ Although implantable CRT devices provide a complete set of pacing options beyond atrio‐biventricular synchronization including rate‐responsiveness (RR), landmark CRT trials were conducted with 30 to 40 bpm lower rate programming to ensure persistent atrial sensing and actual atrial‐tracking (VDD) working mode, as per the EHRA/HRS experts statement.^3^ Bernheim et al highlighted that in CRT patients atrial pacing should be avoided to ensure optimal interatrial and atrio‐ventricular synchrony, therefore the role of atrial pacing and RR function in CRT patients is unclear.^4^ This impacts on optimization of device programming, and on medical therapy titration, since an important target of medical therapy in HF is to lower the heart rate, although chronotropic response may be depressed. Indeed, β‐blockers up‐titration and ivabradine addition to lower the heart rate below 60 bpm at rest is mandatory to reach optimized medical therapy, on top of which CRT is recommended.^5^, ^6^ The prevalence of sinus node disease, that prevents β‐blockers use, in HF patients is extremely low, as to be unreported in CRT landmark trials and in current guidelines.^1^, ^2^, ^6^ One single randomized trial comparing RR atrio‐biventricular pacing to atrial‐tracking modes in unselected CRT recipients yielded neutral results in terms of mortality and HF events,^7^ yet, RR function may benefit specific HF patients' subgroups with very limited heart rate variability and poor rate increase in daily‐life activity,^8^ meaning that this point deserves further investigation. Atrial pacing can prevent atrial fibrillation in sinus node disease patients, whereas no effect on atrial fibrillation has been demonstrated in other settings, as HF. Atrial fibrillation may be a major complication in HF patients, being a well‐known negative prognostic factor associated to increased mortality and HF progression.^9^ Modern technologies of remote monitoring (RM) of implantable devices offer an extremely powerful tool to monitor atrial fibrillation and easily provide a detailed documentation of arrhythmia duration and clinical complications. In fact, RM is recommended for early detection and quantification of atrial fibrillation,^10^ especially in CRT patients because it causes loss of therapy. In our analysis, we used RM in CRT recipients to investigate the association between atrial RR and the incidence of device‐detected atrial arrhythmias, namely, atrial high rate episodes (AHREs). An effort was made to assess whether any observed effect of RR function should be ascribed to atrial pacing itself or to the increased heart rate related to RR.

## 2 METHODS

### 2.1 The Home Monitoring Expert Alliance project

We used the database of the Home Monitoring Expert Alliance (HMEA) project, an independent network of sites with the purpose of establishing a nationwide repository of pooled data generated by RM of cardiac implantable electronic devices during routine practice.^11^ The present analysis was proposed by the corresponding author, reviewed and approved by a seven‐member executive committee from the sites with largest RM data volumes; 41 participating sites (listed in the Appendix) voluntarily contributed to the dataset of this analysis. The HMEA project was approved by Ethics Committees and all patients gave written informed consent for RM and clinical data processing.

### 2.2 Data selection

All RM data were generated by the Home Monitoring system (BIOTRONIK SE KG & Co., Berlin, Germany) a well‐known fully automatic RM technology, characterized by daily device to patient‐unit telemetry, and daily transmissions from patient‐unit to a central Service‐Center through the GSM network for mobile communications. For the purpose of our analysis, we firstly selected 1226 consecutive patients who received an implantable defibrillator with CRT function (CRT‐D) from 2008 to 2017. We further excluded 390 patients due to permanent atrial fibrillation (280), device replacements,^1^1 CRT function programmed off for any reason (144). Eventually, 836 patients provided data that could be processed. Device replacements were excluded to avoid the risk of possible arrhythmic episodes experienced with the previous device and not properly documented.

### 2.3 Analysis endpoints

The primary endpoint of the analysis was time to first post‐implant date with atrial high rate episode (AHRE). The following AHRE burden cutoffs were considered: ≥15 minutes, ≥5 hours, and ≥ 24 hours. AHRE detection was based on the rate criterion with a detection limit of 200 bpm in most cases. In order to correctly evaluate the association between atrial pacing and AHRE incidence, atrial arrhythmic burden was not preferred as study endpoint, as periods of atrial fibrillation should necessary be excluded from the analysis. In fact, atrial fibrillation and atrial pacing are normally inversely related due to the obvious pacing inhibition during the arrhythmia.

The main objective of the analysis was to evaluate whether RR function was associated to an increased or decreased incidence of AHRE. Patients were therefore divided into subgroups according to whether the RR function was programmed on or off. When programmed on, the RR function was provided by standard accelerometers nominally programmed. The analysis was repeated in subgroups of tertiles of baseline mean heart rate. To this end, we excluded the first 30 days post‐implant and calculated the average of daily‐sampled heart rate over the second month post‐implant.

In order to investigate whether the association between RR function and AHRE incidence was mediated by other factors, the following variables were also evaluated: atrial pacing percentage (AP%), CRT percentage (CRT%), and 24‐hour average of heart rate (24 hours). As these data were longitudinal with daily sampling, the variables were included in time‐dependent proportional‐hazard models, averaging over 30‐day periods and adjusting for main baseline variables. These models were used to fit AHRE burden ≥15 minutes and 5 hours. Analysis for AHRE burden ≥24 hours was omitted as pacing percentages and heart rates could have been significantly biased by long‐lasting AHREs.

### 2.4 Statistical analysis

We described the selected population by using RR programming at baseline as grouping criteria. Binary and categorical variables were reported as percentages of available data and compared between groups with the Pearson χ² or Fischer tests, as appropriate. Continuous variable distributions were reported as median (interquartile range) and checked for normality with the Shapiro‐Wilk test. Between‐group comparisons were performed with the Mann‐Whitney *U*‐test if the normality hypothesis could be rejected. Missing data were not replaced; all available data were used for sample distributions evaluation and proportions calculation. RR association with AHRE incidence was evaluated with univariate proportional‐hazard Cox model with the whole population and within each second‐month heart rate tertile, reporting the relative hazard ratio (HR) along with the 95% confidence interval (CI). Schoenefeld's residual method was used to test the proportional‐hazard assumption. Plots of AHRE‐free rates were generated as Kaplan‐Meier curves and the cumulative proportions of patients with AHREs during follow‐up and the CI were calculated with the product‐limit method. Multivariate time‐dependent proportional‐hazard Cox models were also used to separately fit AHRE incidence with AP%, CRT%, and 24 hours as longitudinal variables, taking averages every 30 days. Sex, age, and ischemic cardiomyopathy were used as adjusting baseline variables. All statistical tests were significant with *P* ≤ .05. All analyses were performed with Stata software version 11.1SE (StataCorp, Texas).

## 3 RESULTS

### 3.1 Patient characteristics

Of the 836 CRT‐D patients included in the analysis, 166 (19.8%) had the RR function programmed on at baseline (Table 1). The groups with RR on and off did not show statistically significant differences in any considered characteristics among age, New York Heart Association Class, cardiomyopathy, comorbidities, history of arrhythmias, pharmacological treatment, except for a slightly higher prevalence of RR on in male and diabetes patients. Interestingly, the average basic rate programmed at baseline was higher in the RR on group, 64.3 vs 60.3 bpm in RR off group (*P* < .0001). Excluding the first month post‐implant period for implant stabilization, the 30‐day average AP% at the second month was significantly higher in the RR on group (62.7% vs 11.5%, *P* < .0001), but no differences were detected in CRT% and 24 hours.

**Table 1:** Patient characteristics

		Rate responsive		
	All patients	On	Off	*P*
No. of patients (n, %)	836 (100%)	166 (19.8%)	670 (80.1%)	—
Age (years)	72 (65‐78)	72 (65‐77)	73 (66‐79)	.12
Female (n, %)	205 (24.8%)	29 (17.9%)	176 (26.5%)	.02
NYHA class (n, %)				
I‐II	463 (60.9)	88 (58.6)	375 (61.4)	.62
III‐IV	298 (39.1)	62 (41.4)	236 (38.6)	
*Comorbidities (n, %)*				
Hypertension	383 (56.4)	77 (60.6)	306 (55.4)	.17
Diabetes	186 (28.1)	46 (36.5)	140 (26.2)	.02
COPD	87 (13.8)	20 (15.9)	67 (13.3)	.27
Vascular disease	54 (9.0)	6 (4.8)	48 (10.2)	.06
Chronic kidney disease	108 (16.1)	23 (18.0)	85 (15.6)	.52
Liver disease	17 (2.6)	3 (2.4)	14 (2.6)	.87
Cardiomyopathy (n, %)^a^				
IDCM	315 (48.8)	66 (50.8)	249 (48.3)	.62
NIDCM	300 (44.7)	56 (42.1)	244 (45.3)	.50
Hypertrophic	10 (1.5)	2 (1.6)	8 (1.5)	.97
ARVD	1 (0.2)	0 (0.0)	1 (0.2)	.61
Congenital disease	1 (0.2)	0 (0.0)	1 (0.2)	.61
Rhythm disorders (n, %)				
Secondary prevention	73 (9.2)	17 (11.0)	56 (8.8)	.43
History of AF (paroxysmal or persistent)	94 (12.0)	25 (16.5)	69 (10.9)	.07
Sick sinus syndrome	16 (2.6)	1 (0.8)	15 (3.0)	.15
I degree AVB	52 (7.8)	12 (9.3)	40 (7.5)	.49
II/III degree AVB	35 (5.3)	10 (7.8)	25 (4.7)	.12
Left bundle branch block	412 (54.4)	68 (48.1)	344 (55.6)	.10
LVEF (%)	30 (25‐33)	30 (25‐35)	30 (25–33)	.88
QRS duration (ms)	145 (130‐160)	140 (130‐156)	146 (130–160)	.43
Medications (n, %)				
ACE inhibitors	386 (58.1)	71 (55.0)	315 (58.8)	0.44
Sartans	58 (9.8)	11 (8.9)	47 (10.0)	0.73
Diuretics	544 (81.3)	104 (79.4)	440 (81.8)	0.53
β‐blockers	520 (77.6)	99 (76.1)	421 (78.0)	0.66
Spironolactone	109 (18.2)	22 (18.2)	87 (18.2)	0.99
Calcium antagonists	37 (5.8)	7 (5.7)	30 (5.8)	0.99
Nitrates	61 (9.3)	18 (14.5)	43 (8.1)	0.02
Digitalis	35 (5.5)	3 (2.4)	32 (6.1)	0.10
Ivabradine	39 (6.6)	8 (6.4)	31 (6.6)	0.95
Antiplatelet	294 (44.6)	61 (48.0)	233 (43.7)	0.38
Anticoagulants	166 (25.1)	41 (32.2)	125 (23.4)	0.04
Amiodarone	96 (14.6)	25 (19.8)	71 (13.3)	.06
Programmed basic rate at baseline (bpm)				
Centiles				<.0001
1st	40	50	40	
5th	50	50	40	
10th	50	60	50	
25th	60	60	60	
50th	60	60	60	
75th	60	70	60	
90th	70	75	70	
Average	61.1	64.3	60.3	
Pacing parameters during 2nd month				
Atrial pacing (%)	19.9 (2.7‐55.1)	62.7 (34.8‐84.3)	11.5 (1.5‐38.2)	<0.0001
CRT (%)	98.8 (95.8‐99.9)	98.7 (94.8‐99.7)	98.9 (95.9‐99.9)	0.10
Heart rate (bpm)	70.9 (65.7‐76.4)	71.3 (66.1‐75.6)	70.7 (65.7‐76.6)	0.87

Percentages and distributions were calculated using known non‐missing values.

AF, atrial fibrillation; AHRE, atrial high rate episode; ARVD, arrhythmogenic right ventricular dysplasia; AVB atrio‐ventricular block; COPD, chronic obstructive pulmonary disease; CRT, ardiac Resynchronization Therapy; IDCM: Ischemic Dilated Cardiomyopathy; LVEF: left ventricle ejection fraction; NIDCM: Non‐ischemic dilated cardiomyopathy; NYHA: New York Heart Association; RV: Right Ventricle; TIA: Transient ischemic attack.

Reported cardiomyopathies are not mutually exclusive; ARVD and congenital diseases were present in two patients with NIDCM.

### 3.2 Association between RR and AHRE incidence

After a median follow‐up of 23.9 (12.2‐36.0) months, 507 patients (60.6%) experienced at least one AHRE burden ≥15 minutes.

**Figure 1:**
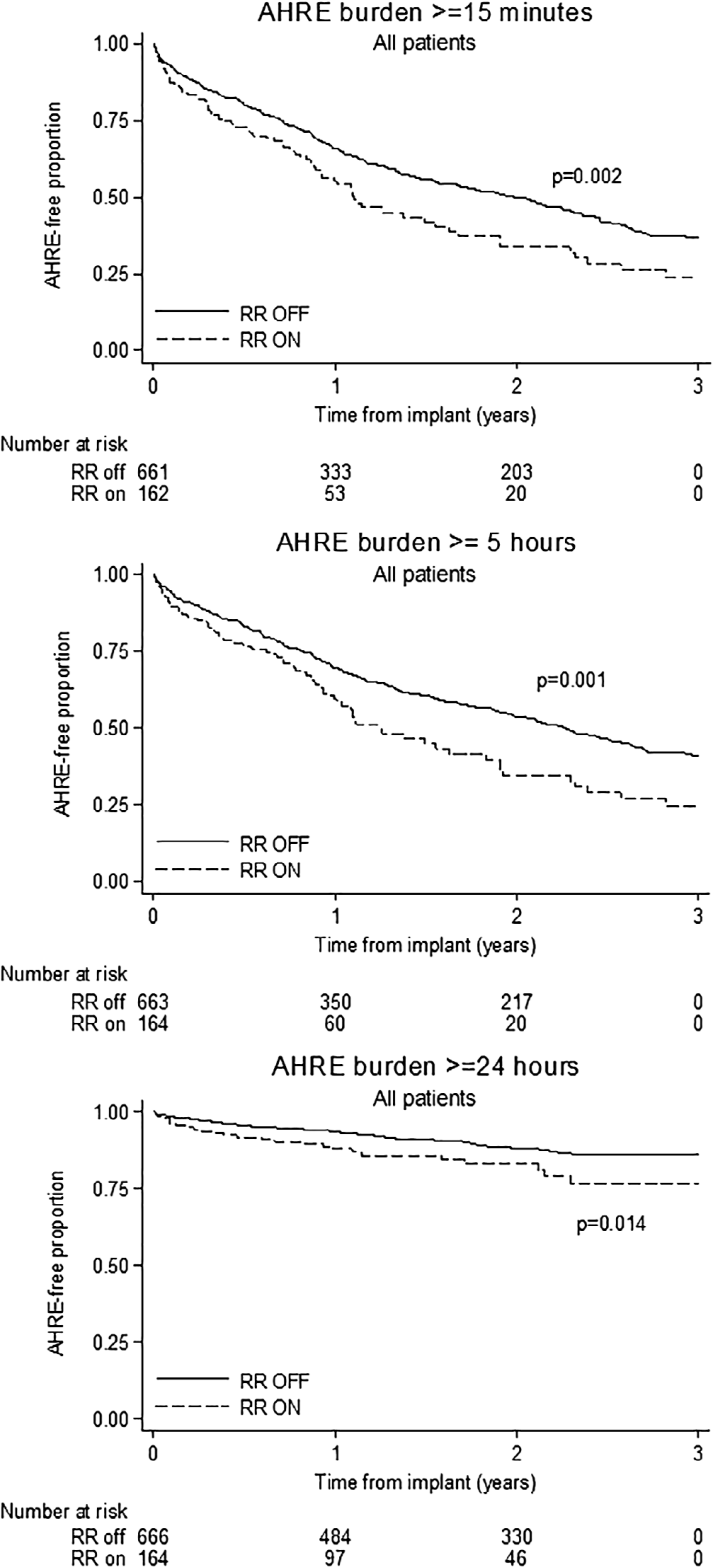
Kaplan‐Meier curves of AHRE burden‐rate using 15‐minute (upper panel), 5‐hour (central panel), and 24‐hour (lower panel) as cutoffs, by RR groups. AHRE, Atrial high rate episodes; RR, rate responsive function

The 3‐year cumulative proportion of patients with AHRE burden ≥15 minutes was 0.76 (CI, 0.65‐0.85) in the RR on group and 0.63 (CI, 0.58‐0.67) in the RR off group, with 1.45 (CI, 1.14‐1.84) HR of RR on vs off (*P* = .002). Figure 1 shows the Kaplan‐Meier curves of AHRE free‐proportion using different cutoffs of AHRE burdens: RR function was also consistently associated to increased risk of AHRE burden ≥5 hours (HR, 1.51, CI, 1.18‐1.94, *P* = .001) and ≥ 24 hours (HR, 1.78, CI, 1.12‐2.82, *P* = .014).

**Figure 2:**
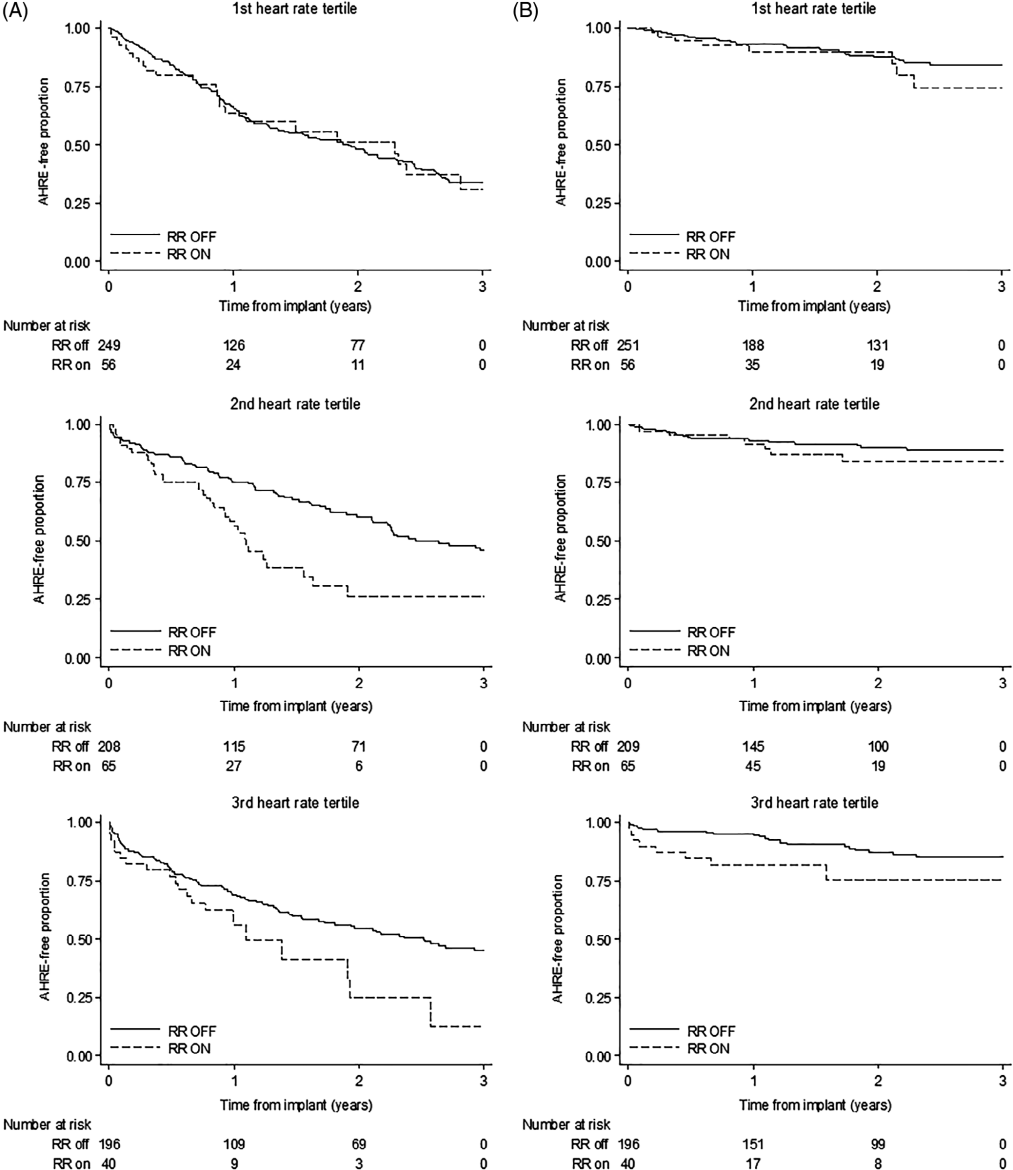
Kaplan‐Meier curve of AHRE burden‐free rates by RR function in each mean heart rate tertile calculated over the second month post‐implant. AHRE burden cutoffs were 5‐hour (A, left graph column) and 24‐hour (B, left graph column). AHRE, Atrial high rate episodes; RR, rate responsive function

The analysis was repeated in each tertile of mean heart rate (first tertile 68.6 bpm; second tertile 74.5 bpm). The HRs and CIs are listed in Table 2. The association of RR function with increased risk of AHRE was either statistically significant or showed a marked trend in the second and third heart rate tertiles. Conversely, no association between RR and AHRE incidence was observed in the first heart rate tertile. Kaplan‐Meier curves with AHRE burden ≥5 and 24 hours in each heart rate tertile are reported in Figure 2.

**Table 2:** AHRE burden Hazard Ratios of RR function on vs off

All patients.			
*AHRE burden*	Hazard Ratio	95% CI	*P*
≥15 minutes	1.45	1.14–1.84	.002
≥5 hours	1.51	1.18–1.94	.001
≥24 hours	1.78	1.12–2.82	.014
1st mean heart rate tertile			
≥15 minutes	1.02	0.67‐1.55	.93
≥5 hours	1.06	0.69‐1.63	.79
≥24 hours	1.56	0.71‐3.42	.27
2nd mean heart rate tertile			
≥15 minutes	2.01	1.37‐2.96	<.001
≥5 hours	1.98	1.32‐2.97	.001
≥24 hours	1.45	0.63‐3.32	.38
3rd mean heart rate tertile			
≥15 minutes	1.50	0.92‐2.44	.10
≥5 hours	1.71	1.04‐2.80	.03
≥24 hours	2.50	1.10‐5.66	.028

*Note*: RR function was associated to an increased risk of AHRE incidence. Mean heart rate was calculated in all patients from 30 to 60 days post‐implant. Sub‐analysis by heart rate tertiles showed that risk was significant in 2nd (≥68.6 bpm) and 3rd HR tertile (≥75.5 bpm) for almost all the selected AHRE burden cutoffs.

AHRE, Atrial High Rate Episode; RR, rate‐responsiveness.

### 3.3 Time‐dependent variables and AHRE incidence

The results of the multivariate Cox analysis including AP%, CRT%, and 24 hours HR as time‐dependent covariates are reported in Table 3 for AHRE burden ≥15 minutes and 5 hours. AP% did not show significant connection with AHREs. Conversely, low amounts of CRT% and, above all, high 24 hours HR were associated with an increased risk of both AHRE burden ≥15 minutes and 5 hours, even after adjusting for the selected baseline characteristics. For unitary decrease in CRT% (*P* < .001) and increase in 24 hours HR (*P* ≤ .013) there was an approximate 2% increase in the risk of AHRE occurrence.

**Table 3:** Time dependent Cox analysis. Association of Atrial Pacing, CRT, 24‐hour heart rate with AHRE incidence

AHRE burden ≥ 15 minutes			
Time dependent covariate Adjusting covariates	Hazard Ratio	95% CI	*P*
AP%	0.997	0.994‐1.002	0.33
Sex (female)	0.746	0.541‐1.028	0.07
Age	1.014	0.999‐1.029	0.06
IDCM	1.223	0.935‐1.600	0.14
CRT%	0.983	0.975‐0.993	.001
Sex (female)	0.756	0.550‐1.039	0.08
Age	1.013	0.998‐1.027	0.08
IDCM	1.210	0.925‐1.581	0.16
Heart rate	1.021	1.004‐1.038	.011
Sex (female)	0.724	0.526‐0.998	0.049
Age	1.017	1.002‐1.032	0.02
IDCM	1.237	0.945‐1.618	0.12
**AHRE burden ≥ 5 hours**			
**Time dependent covariate** **Adjusting covariates**	**Hazard Ratio**	**95% CI**	***P***
AP%	0.998	0.994‐1.003	0.58
Sex (female)	0.711	0.507‐0.996	0.048
Age	1.010	0.995‐1.026	0.16
IDCM	1.179	0.892‐1.558	0.25
CRT%	0.983	0.974‐0.991	<0.001
Sex (female)	0.719	0.515‐1.004	0.053
Age	1.010	0.996‐1.025	0.17
IDCM	1.176	0.891‐1.552	0.25
Heart Rate	1.021	1.005‐1.039	0.013
Sex (female)	0.683	0.488‐0.957	0.03
Age	1.015	0.999‐1.030	0.057
IDCM	1.189	0.907‐1.583	0.20

Multivariate Cox proportional hazard models with time dependent covariates were evaluated to assess the association between AHRE incidence and AP%, CRT% and 24‐hour average HR until time to first AHRE episode.

AHRE: Atrial High Rate Episode; AP%: atrial pacing percentage; CRT%: Cardiac Resynchronization Therapy percentage; HR, heart rate; IDCM ischemic dilated cardiomyopathy.

## 4 DISCUSSION

### 4.1 Main results

In a relatively large population of HF patients implanted with remotely monitored CRT‐D devices as per routine practice, about 50% of devices were programmed with a lower rate of 60 bpm or higher and 20% with RR function on. We observed a 45% to 78% increased risk of both short and long‐lasting AHREs, associated to the activation of the RR function. The negative correlation for the RR function was not observed in the subset of patients with low mean heart rate (<68 bpm). At a multivariate time‐dependent analysis, suboptimal CRT delivery and high mean heart rate were factors significantly associated with AHRE onset. Conversely, we did not find any evidence that atrial pacing itself may affect AHRE incidence.

### 4.2 Role of atrial pacing in the prevention of atrial fibrillation

Several investigations performed in the past provided controversial results about RR function and atrial pacing above intrinsic rate as effective strategies for preventing atrial fibrillation in sinus node dysfunction.^12^, ^13^ The main electrophysiological mechanisms invoked as preventative were the suppression of ectopic activity and the reduction of bradycardia‐induced temporal dispersion of atrial refractoriness. More recently, long‐term heart rate variability has been correlated to atrial fibrillation,^14^ reinforcing the utility of RR pacing in patients with severe sinus bradycardia to help resuming a more physiological heart rate modulation. However, the preventive effect of atrial pacing with or without RR functions has never been definitely assessed and quantified so far. One of the reasons may be merely technical. Atrial fibrillation and atrial pacing are normally inversely related, but this does not necessarily imply any beneficial effect of atrial pacing, due to the obvious pacing inhibition during the arrhythmia. Analyses should carefully exclude periods of atrial fibrillation to correctly evaluate the effect of atrial pacing in preventing episode recurrences. In fact, a high atrial pacing percentage due to RR is associated to long‐lasting AHRE in ICD recipients with ventricular dysfunction.^15^ The result may reasonably raise the question whether atrial pacing itself may be somewhat pro‐arrhythmic, with possible AHRE triggering effects related to pacing in vulnerable periods after undersensing, or poor adaption of atrial refractory period at high heart rate, especially in patients with structural heart disease.^15^ In our analysis, atrial pacing percentage was sampled daily with the used RM system. This enabled us to obtain temporal trends of AP%, rather than cumulative average values. We therefore used time to first AHRE burden date as the response variable and treated AP% as a time‐dependent variable, in order to more accurately reflect the longitudinal data structure and more efficiently process larger amount of the embedded information. This is actually one of main novelties of our study. When modeling atrial pacing and heart rate as time‐dependent covariates, we found no evidence that atrial pacing per se directly promotes AHREs. The results of the analysis reported at the Table 3 (performed independently of the RR function on or off) showed neutral effect of atrial pacing on time to first AHRE onset. Conversely, the association of increased mean heart rate with reduced time to AHRE onset was significant, despite the same available statistical power. Such analysis could not confirm any direct pro‐arrhythmic effect of atrial pacing itself, with a substantially neutral relationship with AHRE onset in our CRT‐D population.

### 4.3 RR pacing in HF

Consistently with previous reports,^15^ we could actually confirm that RR pacing was associated to an increased risk of short‐ to long‐lasting AHREs. Heart rate seems to play the most relevant role, as the association was prominent in the subset of patients with higher (>68 bpm) mean heart rates. This inevitably calls into question the underlying physiopathology of HF, and the beneficial effect of heart rate reduction in the setting of systolic left ventricular dysfunction: in fact, RR pacing conflicts with the main clinical effect of rate‐lowering drugs that have evidence‐based efficacy.^16^ Indeed, cardiovascular mortality and HF events decreased by attaining a < 60 bpm resting rate in the SHIFT study.^5^ Similarly to other reports^17^ around 78% of our patients were treated with β‐blocker therapy at implant: although further up‐titration often occurs during follow‐up,^17^ the specific effect of RR pacing is to offset beta‐blocker therapy by artificially increasing the heart rate, blunting their efficacy. The 2012 experts consensus on CRT clearly recommends to mimic a VDD mode at 35 to 40 bpm lower rate without RR,^3^ to enhance the achievement of a > 50 bpm rate in daily hours. In CRT patients no clinical effect of RR pacing of a high atrial support rate has been reported.^7^ Indeed atrial dyssynchrony may arise with atrial pacing unless an optimized paced AV interval is achieved.^4^ Impaired left ventricular filling translates into increased left atrial stretching, that may in turn promote atrial arrhythmias.^18^ Moreover, RR pacing and a high resting rate decrease heart rate variability, that is a prognostic marker in HF: blunting the rate‐lowering effects of beta‐blockers is associated to worsening of left ventricular (LV) function.^19^ Finally, the increased pacing percentage associated with RR could impact on device longevity, this latter being negatively associated with pacing percentage.^20^ Although chronotropic incompetence is frequently encountered in HF patients either as a pharmacological effect of drugs or as β‐receptors down‐regulation, it is rarely associated to symptoms of reduced exercise tolerance, being the relationship of cardiac output with heart rate flattened at a ceiling heart rate of 100‐110 bpm in systolic LV dysfunction.^21^ These observations make atrial pacing redundant in the majority of CRT recipients, and have prompted the use of two‐leads CRT mimicking VDD mode.^22^

Programming 60 to 70 bpm lower rates and activating the RR function in CRT‐D devices disrupts physiologic heart rate behavior when sinus node performance is still adequate, a significant association with AHRE onset being observed despite relatively small amounts of atrial pacing. RR feature should be reserved for those patients with marked resting bradycardia in the range of 40 bpm and no/little increase during exercise, thus showing reduced heart rate variability and exercise intolerance: in this setting, restoration of a normal rate fluctuation in daily activity may exert symptomatic improvement, and is devoid of unwanted effect on atrial arrhythmias, as our data pointed out (Table 2).

#### 4.3.1 Limitations

The limitations of a registry rather than a randomized study apply to our analysis including the impossibility to test a formal hypothesis. As there are no established indications for the use RR function in sinus rhythm CRT patients, the decision of switching RR on or off was purely based on physicians' personal believes and perceptions. This is a typical trait of large retrospective observations, nevertheless, it represents the main strength stemming from patients' enrolment in real‐life clinical practice. Our study addressed patients in sinus rhythm that represent about 80% of CRT recipients at implantation. Patients in permanent AF or AF as “destination” rhythm are managed differently, and according to current practice they receive either atrio‐ventricular node ablation or strict pharmacologic rate control to ensure CRT delivery as close as possible to 100%. In this setting, RR is often used to achieve a target 100 to 110 bpm to ensure exercise tolerance. Finally, as the HMEA database is based on the Home Monitoring system, all results were obtained with a single manufacturer RR pacing algorithm potentially compromising in some way the extrapolation to other devices.

## 5 CONCLUSIONS

RR pacing in CRT recipients is associated with an increased burden of short‐ to long‐lasting AHREs when the average heart rate exceeds 68 bpm. The extent of atrial pacing per se does not seem to predict the occurrence of atrial arrhythmias, rather the average rate is the strongest AHRE predictor. In this population, a low heart rate seems to confer advantage also in the prevention of atrial arrhythmias, beyond the well‐known effect on HF outcome.

## CONFLICT OF INTEREST

D.G. and A. G. are employees of BIOTRONIK Italia. All other authors have reported that they have no conflicts relevant to the contents of this paper to disclose. This research did not receive any specific grant from funding agencies in the public, commercial, or not‐for‐profit sectors.

## Supporting information


**APPENDIX S1** Supporting information
