# Supplementary material for: Rate‐responsive pacing and atrial high rate episodes in cardiac resynchronization therapy patients: Is low heart rate the key?
Source: Clin Cardiol. 2019 Jul 7;42(9):820–8. doi: 10.1002/clc.23227 (PMC6727874; doi:10.1002/clc.23227)
Supplement: Supplementary file 1 — APPENDIX S1 Supporting information [file CLC-42-820-s001.docx]

# Supplementary material

## List of HMEA centres and investigators.

AOU "G. Martino", Messina: Giuseppe Picciolo, Pasquale Crea; Arcispedale Santa Maria Nuova, Reggio Emilia: Fabio Quartieri, Nicola Bottoni, Matteo Iori; C.D.C. Montevergine, Mercogliano: Francesco Solimene; Carlo Poma, Mantova: Patrizia Pepi, Albino Reggiani; Civile Ferrari, Castrovillari: Giovanni Bisignani; Desio Hospital, Desio: Giuseppe Mantovani, Giulia Balestri; Ferrari, Casarano: Donato Melissano, Giovanni Carlo Piccinini; Ferrarotto, Catania: Valeria Calvi, Francesco Platania; Fondazione di Ricerca e Cura Giovanni Paolo II, Campobasso: Matteo Santamaria, Parisi Quintino, Celestino Sardu, Loredana Messano; FTGM Pisa, Pisa: Marcello Piacenti, Luca Panchetti, Umberto Startari, Andrea Rossi; Gruppo Villa Maria Care&Reserarch, Cotignola: Saverio Iacopino, Pasquale Filannnino, Paolo Artale; INRCA, Ancona: Marinella Marini, Lorenzo Pimpini; Lodi Hospital, Lodi: Fabio Lissoni, Stefano Tinelli, Giulia Caetani, Egidio Marangoni, Sara Morbio; Macerata Hospital, Macerata: Gianluigi Morgagni; Maria Vittoria, Torino: Massimo Giammaria, Claudia Amellone, Maria Teresa Lucciola; Mater Salutis, Legnago: Gabriele Zanotto, Emanuela Visentin, Davide Sandrini; Misericordia, Grosseto: Gennaro Miracapillo, Luigi Addonisio, Marco Breschi, Francesco De Sensi; Monaldi Ospedalieri, Napoli: Antonio D'Onofrio; OO.RR. S.Giovanni di Dio e Ruggi D'Aragona, Salerno: Michele Manzo, Gaetana Melchiorre; S. Croce, Cuneo: Antonello Vado, Gianpaolo Baccari, Cecilia Goletto; Ospedale Apuane, Massa: Giuseppe Arena, Massimo Ratti, Vincenzo Borrello, Iacopo Bertolozzi; Ospedale dei Colli - Monaldi Sun, Napoli: Gerardo Nigro, Ernesto Ammedola, Vincenzo Russo, Anna Rago; Ospedale dell'Angelo, Mestre: Elena Marras, Sakis Themistoclakis; Ospedale di Circolo e Fond. Macchi Cardiologia 1, Varese: Fabrizio Caravati; Ospedale di Circolo e Fond. Macchi Cardiologia 2, Varese: Paolo Bonfanti; Ciriè Hospital, Ciriè: Gaetano Senatore, Giuseppe Trapani, Claudia Amellone, Marco Giuggia; Sacco Hospital, Milano: Giovanni Forleo, Leonida Lombardi; P.O. San Paolo, Bari: Pasquale Cardarola, Luigi Mancini, Cosimo Campanella, Manuela Resta; P.O.S. Timoteo, Termoli: Emilio Musacchio; Policlinico Federico II, Napoli: Antonio Rapacciuolo, Francesca Esposito, Gianluigi Iovino, Alessia Agresta; Pugliese – Ciaccio, Catanzaro: Giampiero Maglia; Riuniti Lancisi, Ancona: Alessandro Capucci; S. Antonio Abate, Gallarate: Daniela Orsida; S. Gerardo, Monza: Giovanni Rovaris; San Filippo Neri, Roma: Carlo Pignalberi, Loredana Morichelli, Antonio Porfili, Laura Quarta, Anna Sassi; San Raffaele, Milano: Paolo Della Bella, Pasquale Vergara, Caterina Bisceglia; Santa Chiara, Trento: Massimiliano Marini, Alessio Coser, Fabrizio Guarracini; Sant'Anna, Como: Stefano Pedretti, Chiara Belvito, Carlo Piemontese; Sant'Orsola, Bologna: Mauro Biffi, Matteo Ziacchi, Igor Diemberger, Cristian Martignani; Spedali Civili, Brescia: Antonio Curnis, Luca Bontempi, Manuel Cerini; Vito Fazzi, Lecce: Ennio C. Pisanò, Tiziana Mazzella.
